# Numerical simulation of frost formation and heat transfer on fin-and-tube heat exchangers in turbulent cross-flow

**DOI:** 10.1098/rsta.2024.0366

**Published:** 2025-07-17

**Authors:** Mahsan Farzaneh, Nadim Zgheib, S. Balachandar, S. A. Sherif

**Affiliations:** ^1^ Department of Mechanical and Aerospace Engineering, University of Florida, Gainesville, FL 32611, USA; ^2^ Department of Mechanical Engineering & Institute for Advanced Manufacturing, The University of Texas Rio Grande Valley, Edinburg, TX 78539, USA

**Keywords:** frost formation, ice physics, heat and mass transfer, fluid mechanics, thermodynamics, refrigeration

## Abstract

Frost formation in fin-and-tube heat exchangers in turbulent cross-flow presents significant challenges in industrial refrigeration applications, affecting heat transfer efficiency and operational reliability. The purpose of this work is to investigate frost deposition and growth on a staggered bank of a fin-and-tube freezer coil under turbulent forced convection conditions. The focus here is on investigating conditions that closely replicate real-world scenarios in large walk-in industrial freezers. Using a direct numerical simulation approach, we examine the flow dynamics and thermal behaviour in the presence of frost, considering turbulent regimes characterized by a Reynolds number in the range $1050\leq Re_{D,\text{avg}}\leq 4800$, with the characteristic length being the outer diameter of the tube and the velocity being the bulk fluid velocity between the plates (fins). Computational fluid dynamics simulations are employed to resolve the interactions between turbulent airflow and the frost layer. Our approach incorporates a modified immersed boundary method and a slow-time acceleration technique to address the complex dynamic interface between the continuously evolving frost layer and the flowing air stream. Our findings indicate that frost forms more on the sides of the finned surfaces (plates) and less on the tubes themselves.

This article is part of the theme issue ‘Heat and mass transfer in frost and ice’.

**Table 1.** Nomenclature.

Latin symbols	
$c_{p,a}^{*}$	specific heat of air, J/(kg·K)
$c_{p,l}$	depth-averaged specific heat of the frost layer, $c_{p,l}=c_{p,l}^{*}/c_{p,a}^{*}$, non-dimensional
$D^{*}$	tube diameter, $D^{*}=5L_{z}^{*}$, m
$D_{x}^{*}$	distance between tube centres in the streamwise direction, $D_{x}^{*}=12.48L_{z}^{*}$, m
$D_{y}^{*}$	distance between tube centres in the cross-flow direction, $D_{y}^{*}=5.24L_{z}^{*}$, m
$f_{u}$	coupling force from the immersed boundary method, non-dimensional
$D_{a}^{*}$	mass diffusion coefficient of water vapour in air, m^2^ s^−1^
$D_{l}^{*}$	lateral mass diffusion coefficient of frost, m^2^ s^−1^
$f_{\theta }$	thermal coupling term from the immersed boundary method, non-dimensional
$f_{w}$	vapour fraction coupling term from the immersed boundary method, non-dimensional
$g_{\theta }$	external thermal forcing term, non-dimensional
$g_{w}$	external vapour fraction forcing term, non-dimensional
$k_{a}^{*}$	thermal conductivity of air, W/(m·K)
$k_{l}$	depth-averaged thermal conductivity of the frost layer, $k_{l}=k_{l}^{*}/k_{a}^{*}$, non-dimensional
*l*_sub_	latent heat of reverse sublimation, $l_{\text{sub}}=l_{\text{sub}}^{*}/(c_{p,l}^{*}(T_{\infty }^{*}-T_{p}^{*}))$, non-dimensional
$L_{x}^{*}$	length of the fins, $L_{x}^{*}=24.96L_{z}^{*}$, m
$L_{y}^{*}$	width of the fins, $L_{y}^{*}=10.4L_{z}^{*}$, m
$L_{z}^{*}$	half of the fin separation, dimensional reference length, m
Nu	Nusselt number as defined in equation (2.10), non-dimensional
$N_{x}$	number of grid points in the x direction, $N_{x}=720$
$N_{y}$	number of grid points in the y direction, $N_{y}=300$
$N_{z}$	number of grid points in the z direction, $N_{z}=201$
$p_{a}^{*}$	partial pressure of dry air, Pa
$p_{f}^{*}$	partial pressure of water vapour over ice, Pa
$Pr_{a}$	Prandtl number of air, $Pr_{a}=\nu_{a}^{*}/\alpha_{a}^{*}$, non-dimensional
R	tube radius, $R=R^{*}/L_{z}^{*}$, non-dimensional
$R_{a}^{*}$	specific gas constant for air, J/(kg·K)
*Re*_*D*,avg_	bulk Reynolds number, $Re_{D,\text{avg}}=U^{*}D^{*}/\nu_{a}^{*}$
${Re}_{\tau }$	shear Reynolds number, ${Re}_{\tau }=U_{\tau }^{*}L_{z}^{*}/\nu_{a}^{*}$
$Sc_{a}$	Schmidt number of air, $Sc_{a}=\nu_{a}^{*}/D_{a}^{*}$, non-dimensional
$S_{f}^{*}$	frost thickness, m
$S_{f}$	frost thickness, $S_{f}=S_{f}^{*}/L_{z}^{*}$, non-dimensional
Sh	Sherwood number as defined in equation (2.10), non-dimensional
t	time, $t=t^{*}U^{*}/L_{z}^{*}$, non-dimensional
$T_{a}^{*}$	air temperature, K
$T_{b}^{*}$	^a^bulk temperature, $T_{b}^{*}=\frac{1}{L_{x}^{*}L_{y}^{*}L_{z}^{*}}\int_{0}^{L_{x}^{*}}\int_{0}^{L_{y}^{*}}\int_{-L_{z}^{*}}^{L_{z}^{*}}T^{*}(x^{*},y^{*},z^{*},t^{*})dx^{*}dy^{*}dz^{*}$, K
$T_{f}^{*}$	frost surface temperature, K
$T_{l}^{*}$	depth-averaged temperature of the frost layer, K
$T_{p}^{*}$	surface temperature of fins and tubes, K
$T_{\infty }^{*}$	^a^reference temperature, $T_{\infty }^{*}=\frac{1}{L_{x}^{*}L_{y}^{*}}\int_{0}^{L_{x}^{*}}\int_{0}^{L_{y}^{*}}T^{*}(x^{*},y^{*},z^{*}=0,t^{*})dx^{*}dy^{*}$, K
u	velocity vector, $\text{u}={\text{u}}^{*}/U^{*}$, non-dimensional
$U^{*}$	bulk velocity, m s^−1^
w	scaled water vapour fraction, non-dimensional
${\tilde{w}}_{a}$	local vapour fraction anywhere in the heat exchanger (coil), kg water vapour/kg dry air
${\tilde{w}}_{f}$	local vapour fraction in the vicinity of the frost surface, kg water vapour/kg dry air
${\tilde{w}}_{\infty }$	reference vapour fraction at the reference temperature, kg water vapour/kg dry air
Greek symbols	
$\alpha_{a}^{*}$	thermal diffusivity of air, m^2^ s^−1^
$\epsilon^{*}$	enhancement factor, non-dimensional
$\theta_{a}$	air temperature, non-dimensional
$\theta_{f}$	frost surface temperature, non-dimensional
$\theta_{l}$	depth-averaged temperature of the frost layer, non-dimensional
$\nu_{a}^{*}$	kinematic viscosity of air, m^2^ s^−1^
$\rho_{a}^{*}$	air density, kg m^−3^
$\rho_{l}$	depth-averaged density of the frost layer, $\rho_{l}=\rho_{l}^{*}/\rho_{a}^{*}$, non-dimensional
τ	slow-time acceleration factor, non-dimensional
ϕ	azimuthal coordinate, rad

^a^ The integral excludes the regions occupied by the tubes and the frost layer.

## 1. Introduction

The formation of frost on the surfaces of heat exchangers presents a significant challenge in industrial refrigeration systems, negatively affecting both the performance and efficiency of the system [1]. Frost forms when moist air comes into contact with a surface whose temperature is equal to or lower than the dew-point temperature of water vapour in air and also below the freezing point. When that happens, reverse sublimation of the water vapour in the air occurs, and the water vapour is deposited onto the surface as frost in a diffusion-driven process. Reverse sublimation is the transformation of vapour to solid, bypassing the liquid phase [2]. If the surface in question is that of a freezer coil, the initial stages of formation may temporarily cause an increase in the heat transfer rate. This is partially due to the increase in the heat transfer area as a result of frost deposition on the coil and partially due to the rougher frost surface that tends to act as micro-fins. As frost builds up further on the coil, the air passages through the coil narrow and the airflow is partially obstructed, resulting in a decrease in the heat transfer rate. If the freezer coil is of the fin-and-tube design, the problem becomes significantly more complex to analyse because of the presence of fins (plates) that cause additional surfaces for the frost to collect on and hence a more complex interaction between the air stream and the finned coil surfaces. Furthermore, frost can act as an insulating layer that causes a further decrease in the heat transfer rate [3–6] (table 1).

Early studies of frost formation in heat exchangers focused primarily on understanding the basic mechanisms of frost growth and its impact on thermal performance. Studies conducted by Niederer [7] and Kondepudi and O’Neal [8–10] offered foundational insights into frost formation on heat exchangers. They found that tightly spaced fins significantly decrease in performance under frosting conditions compared to wider fin spacing. Their research also compared various fin configurations, providing valuable data on how design impacts efficiency in frost-prone environments. Senshu [11] developed an analytical model to predict the frost formation rate in cross-finned-tube heat exchangers and validated their model against published experimental data. Subsequent research expanded on these findings by developing more sophisticated models to predict heat and mass transfer in fin-and-tube heat exchangers under various conditions. Others have explored the influence of different refrigerants and environmental conditions on frost formation. For example, Chen *et al.* [12] investigated how frost deposition on heat exchanger fins affects system performance under forced convection conditions. Yan *et al.* [13] found that increasing the spacing of the fins and adjusting the airflow can help mitigate the negative effects of frost growth in finned-tube heat exchangers. These findings are essential for the design and optimization of heat exchangers operating in frost-prone environments.

Over the years, several models have been proposed to predict frost growth on heat exchanger surfaces. For example, Seker *et al.* [14] developed a detailed model focusing on how frost growth affects heat transfer and the overall efficiency of finned-tube heat exchangers. Their model, coupled with experimental data, has been used to better understand how frost growth changes with environmental factors such as temperature, humidity and airflow. Recent advances in modelling and simulation have made it possible to predict non-uniform frost growth across complex geometries. For example, Padhmanabhan *et al.* [15] developed a semi-empirical model to predict non-uniform frost growth on heat exchangers with improved accuracy. Da Silva *et al.* [16] built on the work of Padhmanabhan *et al.* [15] using a first-principles approach to model frost accumulation in fan-equipped finned-tube evaporators. Their model simulated frost growth over time and predicted how system performance would degrade as frost accumulated.

Frost thickness, fin spacing and air velocity are critical parameters influencing frost formation dynamics, as noted by Lee *et al.* [17], who measured frost growth in round plate finned-tube heat exchangers under varying environmental conditions. Amini *et al.* [18] conducted an experimental study on frost formation in natural convection, highlighting the influence of humidity and temperature on frost growth. Their study resulted in empirical correlations that link environmental factors to frost accumulation.

Wu *et al.* [19] focused on experimental studies of frost formation on cold surfaces with various fin layouts. Their work provided insights into how fin geometry affects frost distribution and the overall heat transfer process. Based on their findings, their later work [1] used computational fluid dynamics to further explore and predict the growth and behaviour of the frost layer in finned-tube heat exchangers, allowing more accurate optimization of heat exchanger designs. Chen *et al.* [20] developed a model showing how frost accumulation reduces airflow and system efficiency, as well as the extent to which it is influenced by factors such as air velocity, temperature and humidity. Zhang *et al.* [21] emphasized the importance of frost distribution along the windward and leeward sides of finned-tube heat exchangers, noting that uneven frost accumulation can severely degrade performance. More recently, Zhang *et al.* [22] examined the effects of frosting conditions on the distribution of frost in air-source heat pump units, revealing how frost accumulates more on windward fins, significantly affecting system efficiency. The work of Dong *et al.* [23] added important data on how frost forms on parallel and counterflow cooling surfaces, showing significant differences in frost characteristics based on flow confinement.

Frost formation in heat exchangers remains a critical challenge in heating, ventilating and air-conditioning (HVAC) and refrigeration applications in general and in industrial refrigeration applications in particular. Although significant progress has been made in understanding frost formation dynamics and in developing better predictive models, more work is needed to optimize system designs and control strategies. Despite these advances, challenges remain, especially in turbulent flow conditions, where frost deposition patterns are significantly more complex. A key computational challenge is the vast difference in time scales between turbulent airflow and frost accumulation. Frost forms slowly at a few millimetres per hour, while turbulent flow evolves in milliseconds, requiring small time steps for accuracy. The small time step coupled with the long integration time makes turbulence-resolving simulations prohibitively expensive. Both airflow and frost also need fine spatial resolution to capture small turbulence structures and the rough surface of the frost. In the present work, we address the issue of vastly different time scales through a slow-time acceleration technique that we have developed and successfully implemented [24–27].

The objectives of the present paper are as follows: (i) demonstrate that two-way coupled turbulence-resolving simulations can effectively address frost formation in complex geometries, such as finned-tube heat exchangers, (ii) investigate the effect of Reynolds number on frost formation in such a complex geometry under turbulent flow conditions and (iii) leverage the time- and spatially resolved temperature and vapour fraction fields to enable us to analyse the temporal and spatial gradients of temperature, frost thickness, as well as Nusselt and Sherwood numbers in the entire system to offer a deeper insight into frost formation in turbulent flow.

Building on these objectives, the present work introduces several novel aspects that distinguish it from previous studies. Specifically, it presents a novel application of two-way coupled numerical simulations to model frost growth on the complex geometry of finned-tube heat exchangers. Unlike previous studies that focused on simpler geometries, such as flat plates or single cylinders, the present study addresses the additional challenges introduced by the staggered tube arrangement of a multi-row deep heat exchanger (freezer coil) and the associated dynamical interactions between the airflow and the frost surface. The methodology leverages mass and energy conservation principles, coupled with a slow-time acceleration technique, to efficiently simulate the time-evolving frost layer. This approach not only captures the key aspects of frost deposition and growth but also provides valuable insights that are hard to obtain experimentally due to the challenges of inserting frost temperature and thickness sensors in the middle rows of the heat exchanger without disrupting the flow. By addressing these challenges, the work helps bridge the gap between experimental limitations and computational modelling, offering a better understanding of frost behaviour in practical engineering scenarios.

## 2. Numerical model

**Figure 1.**
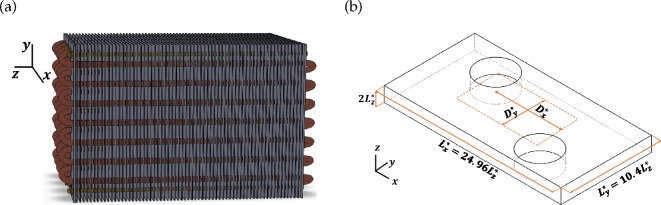
(a) Three-dimensional view of a finned heat exchanger, illustrating the staggered tube and fin arrangement. (b) Schematic of the domain showing two neighbouring plate fins penetrated by two circular tubes in a staggered arrangement. Flow is along the x-axis, and the domain is doubly periodic in the x and y directions to account for the multiple-row nature of this heat exchanger.


A schematic of the geometry considered is shown in figure 1. It consists of two plates (fins) that are separated by a distance $2L_{z}^{*}$ and two cylindrical tubes. The plates (fins) have a length of $L_{x}^{*}=24.96L_{z}^{*}$ and a width of $L_{y}^{*}=10.4L_{z}^{*}$. The two tubes have an outer diameter $D^{*}=5L_{z}^{*}$ and are cut through the plates (fins) in a staggered arrangement as shown in the figure. The centres of the tube are separated by a distance $D_{x}^{*}=12.48L_{z}^{*}$ along the flow (x) direction and by $D_{y}^{*}=5.24L_{z}^{*}$ along the cross-flow (y) direction. The vertical axis (parallel to the (z) direction) of the upstream tube passes through the point $x=L_{x}/4,y=L_{y}/4$*,* and the vertical axis of the downstream tube passes through the point ($x=3L_{x}/4,y=3L_{y}/4$). We solve the following conservation equations of mass (2.1), momentum (2.2) and energy (2.3), as well as the transport equation for the vapour fraction field (2.4)

(2.1)∇⋅u=0(2.2)∂u∂t+u⋅∇u=ex−∇p+1Reτ∇2u+fu.(2.3)∂θa∂t+u⋅∇θa=1ReτPra∇2θa+gθ+fθ.(2.4)∂w∂t+u⋅∇w=1ReτSca∇2w+gw+fw.

In equations (2.1) through (2.4), the length is non-dimensionalized by the half plate separation distance $L_{z}^{*}$, the velocity by the bulk velocity $U^{*}$, the time by the ratio $L_{z}^{*}/U^{*}$ and the pressure by $\rho_{a}^{*}U^{*2}$, where $\rho_{a}^{*}$ is the air density. Throughout this paper, an asterisk signifies a dimensional quantity, and all other quantities are dimensionless. Additionally, the bulk velocity represents the average air velocity within the coil, excluding the tubes and any frost layer that forms on either the tubes or the fins. This is true for any other bulk quantity such as the bulk temperature or the bulk water vapour fraction. The bulk temperature is mathematically defined in table 1 to serve as an example of how a bulk quantity is computed. The air is driven by a mean streamwise pressure gradient, which results in a three-dimensional turbulent flow with velocity field u. The unit vector ${\text{e}}_{x}$ is along the x-direction and corresponds to the mean streamwise pressure gradient, and p is the perturbation pressure. Additionally, $\theta_{a}(x,y,z,t)$ and w(x,y,z,t) represent the non-dimensional air temperature and the scaled water vapour fraction, which are defined as follows:


(2.5) $$\theta_{a}=\frac{T_{a}^{*}-T_{p}^{*}}{T_{\infty }^{*}-T_{p}^{*}},\;\;w=\frac{{\tilde{w}}_{a}-{\tilde{w}}_{p}}{{\tilde{w}}_{\infty }-{\tilde{w}}_{p}},$$

where the temperature scale is set as the difference between the reference temperature, $T_{\infty }^{*}$, and the surface temperature, $T_{p}^{*}$, which is taken to be a constant. The reference temperature represents the average temperature in the plane midway between the two fins, excluding the tubes and the frost layer around the tubes. It is important to note here that $T_{p}^{*}$ is the surface temperature of both the tubes and the fins. While typically there is a temperature gradient along the fin, we believe that the simplifying assumption made here is acceptable given the relatively small temperature change along the fin due to the high thermal conductivity of both the fin material (typically aluminium) and tube material (typically copper). The quantity $T_{a}^{*}$ represents the local dimensional air temperature anywhere in the flow field. Similarly, the water vapour fraction scale is set as the difference between the reference vapour fraction, ${\tilde{\text{w}}}_{\infty }$, and the surface vapour fraction, ${\tilde{\text{w}}}_{p}$. The vapour fraction is typically referred to in psychrometric theory as the humidity ratio. So, the term ‘surface water fraction’ refers to the humidity ratio of the air in the vicinity of the surface. The quantity ${\tilde{\text{w}}}_{a}$ represents the unscaled local air vapour fraction (or local humidity ratio) anywhere in the flow field. The unit of the humidity ratio is kg of water vapour per kg of dry air.

The quantity $Re_{\tau }$ is the shear Reynolds number, which is based on the shear velocity, half the fin-to-fin separation distance and the kinematic viscosity of the air $\nu_{a}^{*}$. Here, ${\text{f}}_{u}$ is the coupling force of the immersed boundary method, which ensures the maintenance of no-slip and no-penetration conditions at the frost surface [28,29] using Uhlmann’s direct forcing method [30]. The Prandtl number of air is defined as $Pr_{a}=\nu_{a}^{*}/\alpha_{a}^{*}$, where $\alpha_{a}^{*}$ is the thermal diffusivity of the air. In the energy equation, $f_{\theta }$ is the coupling term of the immersed boundary method, enforcing the desired temperature at the frost surface. Furthermore, the external thermal forcing term, $g_{\theta }$, is used to maintain the reference temperature at the desired reference value (i.e. at all times $T_{\infty }^{*}=295.15$ K). This enables the application of periodic boundary conditions along the streamwise direction. The term $Sc_{a}=\nu_{a}^{*}/D_{a}^{*}$ represents the Schmidt number, where $D_{a}^{*}$ is the coefficient of mass diffusion (diffusivity) of water vapour in air. Similar to the energy equation, the term $f_{\text{w}}$ enforces the boundary condition at the frost surface, while the forcing term $g_{\text{w}}$ ensures that the reference vapour fraction is maintained at the reference value.

Equations (2.1) through (2.4) are solved using a pseudo-spectral code, which has been validated and applied in similar studies of flow over spatially and temporally evolving surfaces [25,26,31–33]. In this pseudo-spectral code, Fourier expansions are applied to the flow variables in the homogeneous x and y directions. In the inhomogeneous z direction, a Chebyshev expansion is employed with Gauss–Lobatto quadrature points. Time integration of the flow field is performed using a Crank–Nicolson scheme for the diffusion terms. The advection terms are discretized using the Arakawa method and advanced with a low-storage, third-order Runge–Kutta scheme. The time step is chosen to maintain the Courant number below 0.5 for the duration of each simulation.

The airflow occupies the region between the plates (fins), excluding the interior of the cylindrical tubes and the frost layer that develops over time. The domain is assumed to be periodic in the streamwise and spanwise directions, so the simulated portion of the finned-tube heat exchanger corresponds to the fins and tubes located in the interior, away from the inlet and outlet.

The simulations being considered are two-way coupled, meaning the air and frost phases dynamically influence each other. In each simulation, the air phase is resolved using the continuity equation, the Navier–Stokes equation, the energy equation and the water vapour species transport equation. The primary goal of solving these equations is to obtain surface gradients of temperature and water vapour concentration. These gradients are then used in the mass and energy balance equations (2.6)–(2.9) for the frost build-up on the fins and tubes to determine the frost thickness and temperature distribution. As the frost profile evolves over time, this updated information is fed back into the air-phase model via the immersed boundary method by modifying the surface geometry and thermal boundary conditions. This coupling process is repeated for the entire duration of the simulation. As frost accumulates, the available volume for airflow decreases, mimicking the behaviour observed in real fin-and-tube heat exchangers.

The frost, which accumulates on the fins and tubes, is defined by its time-dependent non-dimensional thickness, $S_{f}$, which also depends on the location on the surface. The non-dimensional frost surface temperature, $\theta_{f}$, and the non-dimensional vapour mass fraction, ${\text{w}}_{f}$, are also important quantities that depend on time and location on the surface. In the present problem, the surface dependence of these quantities can be expressed in terms of two space variables, depending on whether we are dealing with the fin or the tube surface. For the fins, they are expressed in terms of the coordinates x and y, such that $S_{f}=S_{f}(x,y,t)$*,* $\theta_{f}=\theta_{f}(x,y,t)$ and ${\text{w}}_{f}={\text{w}}_{f}(x,y,t)$. For the tubes, they are expressed in polar coordinates as $S_{f}=S_{f}(\phi ,z,t)$*,* $\theta_{f}=\theta_{f}(\phi ,z,t)$ and ${\text{w}}_{f}={\text{w}}_{f}(\phi ,z,t)$. The angle ϕ, which varies between 0 and 2π, represents the azimuthal coordinate for the tubes, where ϕ=0 is aligned with the direction of the cross-flow (i.e. the y direction).

The frost growth equations are based on the mass and energy balance of the frost layer. Following the formulation of [25,26], in Cartesian coordinates, for the fins, the conservation of mass (2.6) and energy (2.7) of the frost layer are expressed as


(2.6) $$\begin{array}{lcr} & \frac{1}{\tau }\frac{\partial \left(\rho_{l}\;S_{f}\right)}{\partial t}=\frac{\left({\tilde{\text{w}}}_{\infty }-{\tilde{\text{w}}}_{f}\right)}{Re_{\tau }\;Sc_{a}}Sh+\frac{1}{Re_{\tau }\;Sc_{l}}\nabla^{2}\left(\rho_{l}\;S_{f}\right). & \end{array}$$

(2.7) $$\begin{array}{rl} \frac{\rho_{l}\;S_{f}}{\tau }\frac{\partial \theta_{l}}{\partial t}+\frac{\theta_{l}-\theta_{f}}{\tau }\frac{\partial \left(\rho_{l}\;S_{f}\right)}{\partial t}= & \frac{1-\theta_{f}}{Re_{\tau }\;Pr_{a}c_{p,l}}Nu+\frac{l_{\text{sub}}({\tilde{\text{w}}}_{\infty }-{\tilde{\text{w}}}_{f})}{Re_{\tau }\;Sc_{a}}Sh\\ & -2\frac{k_{l}}{c_{p,l}}\frac{\theta_{l}-\theta_{p}}{S_{f}\;Re_{\tau }\;Pr_{a}}+\frac{k_{l}\;S_{f}}{Re_{\tau }\;Pr_{a}\;c_{p,l}}\nabla^{2}\theta_{l}\;. \end{array}$$

In the above-given equations, $Sc_{l}=\nu_{a}^{*}/D_{l}^{*}$, $\rho_{l}=\rho_{l}^{*}/\rho_{a}^{*}$, $k_{l}=k_{l}^{*}/k_{a}^{*}$, $c_{p,l}=c_{p,l}^{*}/c_{p,a}^{*}$ and $l_{\text{sub}}=l_{\text{sub}}^{*}/[c_{p,l}^{*}(T_{\infty }^{*}-T_{p}^{*})]$, where $D_{l}^{*}$ is the lateral mass diffusivity of frost. Here, $\rho_{l}^{*}$, $k_{l}^{*}$ and $c_{p,l}^{*}$ represent the frost density, frost thermal conductivity and frost-specific heat averaged over the depth of the frost layer. Also, $l_{\text{sub}}^{*}$ is the latent heat of reverse sublimation from water vapour to frost, and $c_{p,a}^{*}$ and $k_{a}^{*}$ represent the air-specific heat and thermal conductivity, respectively. In the mass balance equation, the first term on the right-hand side accounts for frost deposition based on the local mass transfer and the second term corresponds to lateral diffusion of the mass, where $\nabla^{2}$ is the two-dimensional Laplacian along the two lateral directions (upon which $S_{f}$ is dependent).

It is important to note here that frost formation occurs at a rate of millimetres per hour, whereas turbulent flow evolves on a scale of milliseconds. This significant time disparity makes high-fidelity simulations computationally prohibitive for extended durations. To address this challenge, a slow-time acceleration factor τ is applied to the governing equations related to frost, scaling the time step for the frost deposition processes without compromising the fidelity of the underlying flow dynamics. In this approach, the evolution equations of the temperature and thickness of the frost are accelerated by the factor τ. In the present simulations, we have set τ=1000 [25,26].

In the frost energy equation (equation (2.7)), $\theta_{l}$ represents the temperature of the non-dimensional depth-averaged frost layer, such that $\theta_{f}=2\theta_{l}-\theta_{p}$ is the frost surface temperature. The two terms on the left-hand side of equation (2.7) correspond to the energy associated with the increase in the depth-averaged temperature of the frost layer and the energy associated with the newly deposited frost, respectively. On the right-hand side, the four terms correspond to (i) convective heat transfer from the air to the frost layer, (ii) energy addition due to mass exchange from the air stream, (iii) conductive heat loss to the fin surface and (iv) diffusive transport of heat along the x and y directions within the frost layer (again $\nabla^{2}$ is the two-dimensional Laplacian).

On the cylindrical tubes, the frost layer mass and energy balance equations are expressed in polar coordinates as


(2.8) $$\begin{array}{lcr} & \frac{1}{\tau }\frac{\partial \rho_{l}\;S_{f}}{\partial t}=\frac{\left({\tilde{\text{w}}}_{\infty }-{\tilde{\text{w}}}_{p}\right)}{Re_{\tau }Sc_{a}}Sh+\frac{1}{Re_{\tau }Sc_{l}}\frac{R+\frac{1}{2}S_{f}}{R+S_{f}}\left[\frac{1}{{\left(R+\frac{1}{2}S_{f}\right)}^{2}}\frac{\partial^{2}\rho_{l}\;S_{f}}{\partial \phi^{2}}+\frac{\partial^{2}\rho_{l}\;S_{f}}{\partial z^{2}}\right] & \end{array}$$

(2.9) $$\begin{array}{rl} & \frac{1}{\tau }\left[\left(\frac{R+\frac{1}{2}S_{f}}{R+S_{f}}\right)\rho_{l}S_{f}\frac{\partial \theta_{l}}{\partial t}+(\theta_{l}-\theta_{f})\frac{\partial \rho_{l}S_{f}}{\partial t}\right]=\;\frac{(1-\theta_{f})\;Nu}{Re_{\tau }\;Pr_{a}\;c_{p,l}}+\frac{l_{\text{sub}}({\tilde{w}}_{\infty }-{\tilde{w}}_{f})\;Sh}{Re_{\tau }\;Sc_{a}}\\ & \;-\frac{2R}{R+S_{f}}\frac{k_{l}(\theta_{l}-\theta_{p})}{c_{p,l}\;Re_{\tau }\;Pr_{a}\;S_{f}}+\frac{R+\frac{1}{2}S_{f}}{R+S_{f}}\frac{k_{l}S_{f}}{c_{p,l}\;Re_{\tau }\;Pr_{a}}\left[\frac{1}{(R+\frac{1}{2}S_{f}{)}^{2}}\frac{\partial^{2}\theta_{l}}{\partial \phi^{2}}+\frac{\partial^{2}\theta_{l}}{\partial z^{2}}\right], \end{array}$$

where R is the non-dimensional radius of the tube. The above-given equations for the tube are the same as those for the fins, and the scale factors involving R are to take care of the curvature of the cylindrical surface. The Sherwood (Sh) and Nusselt (Nu) numbers in equations (2.6)–(2.9) represent the gradients of vapour fraction and temperature on the frost surface, respectively. They are defined as


(2.10) $$Sh=\frac{1}{{\tilde{w}}_{b}-{\tilde{w}}_{f}}\nabla \tilde{w}\cdot e_{n},\;Nu=\frac{1}{1-\theta_{f}}\nabla \theta_{a}\cdot e_{n},$$

where the gradients $\nabla \tilde{\text{w}}$ and $\nabla \theta_{a}$ are evaluated at the various frost surfaces, and ${\text{e}}_{n}$ is the unit normal vector, which predominantly points vertically upward for the bottom frost surface, vertically downward for the top frost surface and radially outward for the tubes.

The thermodynamic properties involved in equations (2.6)–(2.9) are primarily parameterized as functions of the frost surface temperature, based on empirical relations from [34], which are further detailed in [25,26]. To accurately simulate frost growth, it is essential to use reliable thermodynamic, thermophysical and transport properties for both air and frost. For the air layer, the thermodynamic and transport properties (i.e. $\rho_{a}^{*}$, $k_{a}^{*}$, $\alpha_{a}^{*}$ and $C_{p,a}^{*}$) are assumed to be constant values evaluated at the reference air temperature ($T_{\infty }^{*}$) and standard atmospheric pressure. The kinematic viscosity of air is fixed at $\nu_{a}^{*}=1.42\times 10^{-5}$ m^2^ s^−1^.

While air properties are assumed constant, frost properties vary with temperature, impacting its density, thermal conductivity and specific heat [35]. For frost, empirical relations are used to define its properties. The specific heat $c_{pl}^{*}$ is given by [36]


(2.11) $$\begin{array}{lcr} & c_{pl}^{*}=\frac{1}{\rho_{l}^{*}}(1.85\times 10^{5}+6.89\times 10^{4}T_{l}^{*}), & \end{array}$$

while the frost density $\rho_{l}^{*}$ is calculated using the equation provided by Hayashi *et al.* [37]


(2.7) $$\begin{array}{lcr} & \rho_{l}^{*}=650\;\exp\;\left[0.227(T_{l}^{*}-273.15)\right]\;, & \end{array}$$

where $T_{l}^{*}$ is the dimensional depth-averaged frost layer temperature, which is taken to be the average of the frost surface and plate temperatures. The latent heat of sublimation $l_{\text{sub}}^{*}$ and thermal conductivity $k_{l}^{*}$ are defined based on the density of frost. The latent heat of sublimation ($l_{\text{sub}}^{*}$) is calculated using a modified version of the Parish correlation [38], as described by Mago & Sherif [35], and the thermal conductivity ($k_{l}^{*}$) is defined by Yonko & Sepsy [39]. To calculate the vapour mass fraction at the frost surface, ${\tilde{\text{w}}}_{f}$, the following equation is used [35]:


(2.13) $$\begin{array}{lcr} & {\tilde{\text{w}}}_{f}=\epsilon^{*}p_{f}^{*}/p_{a}^{*}. & \end{array}$$

The enhancement factor $\epsilon^{*}$ accounts for deviations from ideal gas behaviour in the water vapour/air mixture by adjusting the predicted properties of moisture at the frost surface. Olivieri *et al.* [40] originally introduced a logarithmic formulation for $\epsilon^{*}$, capturing the effects of intermolecular forces and pressure variations. Mago & Sherif [35] extended this work by numerically solving $\epsilon^{*}$ over a temperature range of −60°C to 60°C and fitting the results into a polynomial representation. The polynomial approach is computationally efficient and adequately captures the temperature dependence of $\epsilon^{*}$, making it better suited for our numerical approach here. The details of the enhancement factor and the partial pressure of water vapour over ice $p_{f}^{*}$ are provided in appendix A.

## 3. Results and discussion

We conducted three simulations, all of which share the same geometry as shown in figure 1. In all simulations, the fins and tubes are kept at a temperature $T_{p}^{*}=263$ K, while the bulk air temperature is maintained at $T_{b}^{*}=T_{\infty }^{*}=295$ K with a relative humidity of 50%. The exact mathematical definition of the bulk temperature is provided in table 1. The assumption of uniform fin-and-tube temperature is based on the convection-dominated nature of the system, where the temperature differences within the tube and/or fin are negligible compared to the temperature gradients in the airflow and frost. The high thermal conductivity of typical materials used for fins (aluminium) and tubes (copper) further minimizes temperature gradients along the metal surfaces of both. While the surface metal temperature for all cases considered was set at $T_{p}^{*}=263$ K, we anticipate that a lower surface temperature would lead to steeper gradients in both the surface temperature and water vapour concentration. Consequently, we expect a faster frost growth rate and a greater steady-state frost thickness at lower surface temperatures and vice versa. Additionally, we note that under transient conditions, the surface temperature is likely to increase over time. However, in convection-dominated heat transfer, the transient phase is generally short-lived, and steady-state conditions are expected to dominate during most of the frost accumulation period. This supports the current assumption of a constant temperature across the fins and the tubes.

The resolution of the grid for all simulations has been kept the same, with the number of points in the grid along the x, y and z directions being $N_{x}=720$, $N_{y}=300$ and $N_{z}=201$ grid points, respectively. The Prandtl and Schmidt numbers are also the same in the simulations with values of Pr=0.71 and Sc=0.66. However, the simulations only differ by the bulk Reynolds number, which was set at $Re_{D,\text{avg}}=1050$ (*case 1*), $Re_{D,\text{avg}}=2250$ (*case 2*) and $Re_{D,\text{avg}}=4800$ (*case 3*). Each of the aforementioned three cases is run until a stationary state is reached before the frost is allowed to deposit on the surfaces. The bulk Reynolds number, $Re_{D,\text{avg}}$, is defined using the outer diameter of the tube as the characteristic length and the bulk fluid velocity in between the plates (fins) as the velocity.

To address the adequacy of the numerical grid, we conducted an additional simulation at $Re_{D,\text{avg}}=1050$ and with identical conditions to the previous simulations, except that the resolution is increased by 33% in each direction, namely $N_{x}=960$, $N_{y}=400$ and $N_{z}=267$. The higher resolution simulation is allowed to reach a stationary state without any frost accumulation, and the temperature gradients at the tubes and fins are measured and compared against the coarser grid resolution. We observe the approximate relative error in the temperature gradient at both the fins and the tubes to differ by less than 2%. We deemed the result to be satisfactory and adopted a grid resolution corresponding to $N_{x}=720$, $N_{y}=300$ and $N_{z}=201$ throughout the manuscript.

### (a) Air phase

**Figure 2.**
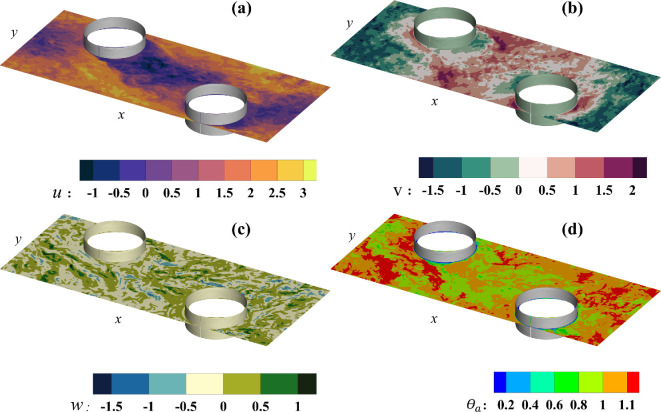
Contours of (a) u, (b) *v* and (c) w, the x, y and z components of velocity, and (d) $\theta_{a}$, the frost surface temperature. The contours are shown in the x–y plane midway between the two fins from case 3 at τt=1500.

Figure 2 shows the instantaneous contours of u (panel a), v (panel b), w (panel c) and $\theta_{a}$ (panel d), which correspond to the x, y and z components of velocity and air temperature, respectively. The contours of the water vapour fraction are qualitatively and quantitatively similar to those of the temperature since the boundary conditions and flow conditions are similar, and both variables are passive scalars that are being advected by the turbulent airflow. The contours correspond to case 3 and are depicted in a horizontal plane located midway between the two plates (fins), where the flow is directed from left to right along the positive x-axis. The plane midway between the two plates is treated as a plane of statistical symmetry. In other words, while local and instantaneous variations may occur, the time-averaged results are expected to exhibit symmetrical behaviour. Figure 2 emphasizes the turbulent nature of the flow and the development of recirculation zones on the leeward side of the tubes, as indicated by the negative streamwise velocity component in panel a (represented by the black and purple contours) extending to the edge of the downstream tube. The magnitude of the cross-flow velocity component is comparable to the streamwise component, as the flow zigzags between the tubes while moving downstream. Furthermore, the alternating positive and negative contours of w in the mid-plane suggest a strong interaction and mixing between the upper and lower regions of the domain. As passive scalars, both the temperature and the water vapour fraction are strongly affected by the flow field and boundary conditions. Typically, air temperature and water vapour fraction are higher upstream of the tubes and lower in their wake. This suggests that frost formation will occur more quickly (due to the higher water vapour fraction) and more densely (due to the higher temperature) on the windward side of the tubes compared to the leeward side.

### (b) Frost phase

**Figure 3.**
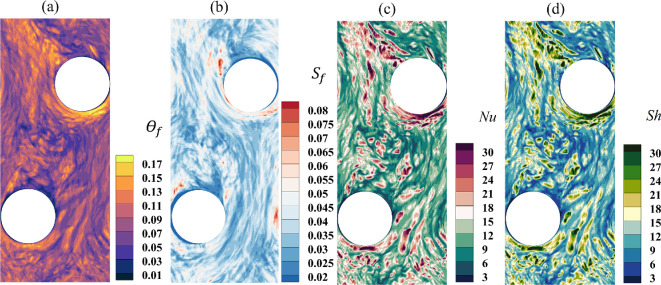
Contours of (a) frost surface temperature, (b) frost thickness, (c) Nusselt number and (d) Sherwood number on the bottom fin at τt=1500 for case 3. Results on the top fin show similar trends both qualitatively and quantitatively.

Frost formation is influenced by the surface temperature and the thickness of the frost layer itself, along with the surface gradients of temperature and water vapour fraction represented by the Nusselt and Sherwood numbers, respectively. Figure 3 shows the contours of the following four quantities: frost surface temperature (panel a), frost thickness (panel b), Nusselt number (panel c) and Sherwood number (panel d) on the bottom fin at τt=1500 from case 3. The strong dynamical interaction between airflow and the surface is evident. The Nusselt and Sherwood numbers range from approximately 3 to 30, where lower values correspond to reduced heat and mass transfer rates, respectively. Larger values are typically observed upstream of the tubes; however, due to the turbulent nature of the flow, instantaneous values can fluctuate. Frost thickness and surface temperature on the fins also exhibit significant variations. Although the frost is still in the early stages of deposition at the time shown, we observe an approximately order-of-magnitude difference in both frost thickness and temperature gradients. The dimensionless frost thickness shown in figure 3 is such that it is directly proportional to the dimensional frost thickness following a straightforward scaling, whereby the half fin-to-fin separation distance is chosen as a length scale. A dimensionless temperature value of 0 represents the fin temperature, $T_{p}^{*}=263$ K, while a value of 1 corresponds to the bulk temperature, $T_{b}^{*}=295$ K (see mathematical definition of the bulk temperature in table 1). Any value between 0 and 1 indicates an increase in the frost surface temperature beyond the fin temperature, with the computed temperature being calculated by multiplying the dimensionless value by $T_{b}^{*}-T_{p}^{*}$. The frost surface temperature along the fins does vary by a few degrees, and at the instant shown, the largest difference is around 4.5 K. This 4.5 K difference reflects the current conditions and is expected to change under different isothermal surface conditions. Nonetheless, temperature variations arise from the combined effects of convective heat transfer to the surrounding air, latent heat release during frost formation and thermal conduction through the frost layer. In other words, the frost surface temperature is influenced by conduction from the plate and tube temperatures. However, because the overall heat transfer is dominated by convection, the impact of frost surface conditions is expected to be minimal.

**Figure 4.**
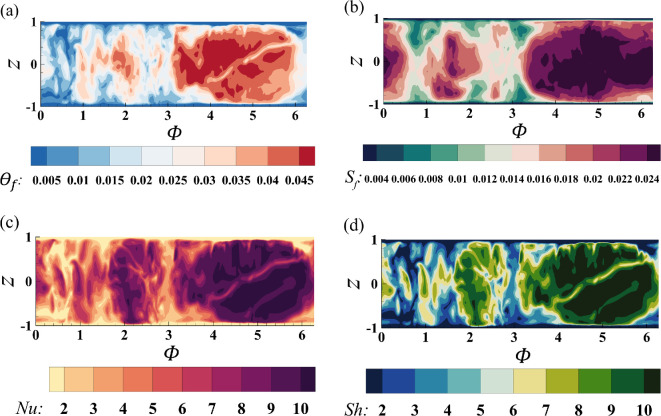
Contours of (a) frost surface temperature, (b) frost thickness, (c) Nusselt number and (d) Sherwood number on the upstream tube at τt=1500 for case 3.

Frost concurrently accumulates on the tubes, albeit at a much slower rate, as shown in figure 4, which corresponds to the upstream tube from case 3 at the same instant of time τt=1500 shown in figure 3 for comparison. In the figure, ϕ corresponds to the azimuthal location, which varies from 0 to 2π, and z represents the vertical location, with z=0 denoting the horizontal x–y symmetry plane midway between the two fins. This slower frost build-up on the tubes is a result of the lower water vapour fraction gradient at the surface, represented by the Sherwood number. As shown in panel d, the Sherwood number varies over a range that is two to three times smaller than that observed on the fins. The same holds true for the Nusselt number.

In the figure, the azimuthal angle ϕ is oriented such that ϕ=π/2 and ϕ=3π/2 correspond to the leeward and windward sides of the tube, respectively. The largest values of Nu and Sh are observed on the windward side, as evidenced by the dark purple (panel c) and dark green (panel d) contours. In addition to the edges of the tube, we observe little variation along the z direction for all variables considered, indicating that frost builds up fairly uniformly along the length of the tubes, except near where they are attached to the fins. However, there is a strong azimuthal variation, as noted earlier. Since both Nu and Sh are about two to three times smaller compared to those on the fins, the frost surface temperature, $\theta_{f}$, and frost thickness, $S_{f}$, are also similarly reduced. In fact, both the non-dimensional frost surface temperature and non-dimensional frost thickness are approximately two to three times smaller than the corresponding values on the fins.

This behaviour is consistent with experimental observations. For example, in the experiments conducted by Zhang *et al*. [22] on frost formation in finned-tube heat exchangers, frost developed differently on the fins compared to the tubes. The frost thickness on the fins was found to increase, particularly on the windward side. The final frost thickness on the fins was greater than that on the tubes. In particular, the windward side of the heat exchanger experienced thicker frost accumulation compared to the leeward side. To further validate our results, we compared the computed Nusselt number with the data reported in [41,42]. Their findings were presented in terms of the Colburn factor (j), which relates the Nusselt number, Reynolds number and Prandtl number through the following equation:


(3.1) $$j=\frac{Nu}{Re_{D,\text{avg}}Pr^{1/3}}.$$

According to those two studies, the Colburn factor for $Re_{D,\text{avg}}=1050$ was approximately j≈0.0084. Using the above-given relationship, this corresponds to a Nusselt number of approximately Nu=7.9. On the other hand, we obtain a Nusselt number of Nu≈6 on the fins and Nu≈4 on the tubes, giving an overall Nusselt number of around 5.3. While the studies reported in [41,42] correspond to a staggered fin-and-tube heat exchanger, the geometry exhibits differences whereby in [41], $7.1≲D_{x}≲14.8$, $5.4≲D_{y}≲11.3$ and 2.9≲D≲6.1, compared to the present values of $D_{x}=12.48$, $D_{y}=5.24$ and D=5.

### (c) Leveraging the eightfold symmetry

With the present assumption of streamwise and spanwise periodicity, the finned-tube geometry exhibits eightfold symmetry for both fins and tubes, allowing eight distinct realizations to be extracted from each simulation. In particular, each fin can be split into four equal regions of size $l_{x}\times l_{y}$ extending along the full length of the fin and a quarter of its width, i.e. $l_{x}=L_{x}$ and $l_{y}=L_{y}/4$. Regions 1 ($0\leq y\leq l_{y}$) and 2 ($l_{y}\leq y\leq 2l_{y}$) are statistically symmetric about $y=l_{y}$, and regions 3 ($2l_{y}\leq y\leq 3l_{y}/2$) and 4 ($3l_{y}/4\leq y\leq 4l_{y}$) are statistically symmetric about $y=2l_{y}$. Furthermore, regions 2 and 3 are symmetric about $y=2l_{y}$, provided the regions are cyclically shifted by a total distance of $D_{x}$ (figure 1) along the flow direction such that the axes of the two tubes are aligned along the x direction. Additionally, the top and bottom fins are statistically similar. Similarly, the tubes also exhibit an eightfold symmetry.

**Figure 5.**
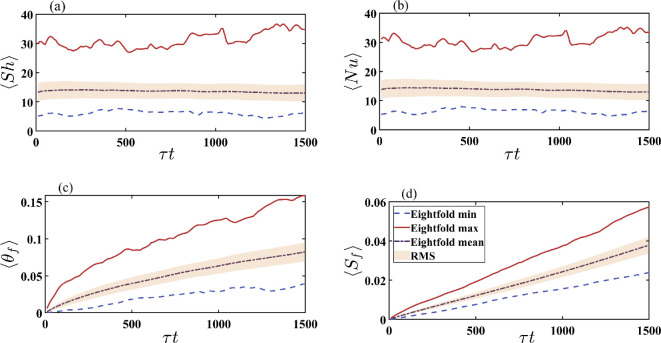
Minimum, maximum, mean and RMS values from the eightfold average on the fins for (a) Sherwood number, (b) Nusselt number, (c) frost surface temperature and (d) frost thickness. τ=1000 is the slow-time acceleration factor, and t represents the non-dimensional time.

This symmetry is exploited to analyse the frost layer’s statistical behaviour. In figure 5, we present the temporal evolution of the eightfold average for the fins in case 3, including the overall values of the average, maximum, minimum and root mean square value (RMS). The quantities of interest are the Sherwood number (panel a), Nusselt number (panel b), surface temperature (panel c) and frost thickness (panel d). The eightfold average, which is the same as the average over the two fins, is shown as a dashed black line surrounded by a shaded region, which corresponds to the RMS. The eightfold average maximum and minimum values are shown as a solid red line and a dashed blue line, respectively.

The Nusselt and Sherwood numbers behave similarly due to the analogous scaling in equation (2.10). The average values of Nu and Sh are about 14, whereas the maximum and minimum values fluctuate around 30 and 3, respectively, with the maximum value exhibiting the largest fluctuations. High values correspond to higher frost deposition rates (Sherwood number) and higher convective heat transfer rates (Nusselt number). The RMS value for Nu and Sh remains approximately 30% of the average throughout the simulation. The near-steady value of Nu and Sh for the duration of the simulation results in a steady increase in the temperature of the frost surface and thickness of the frost layer. However, the variation in both $\theta_{f}$ and $S_{f}$ increases with time, as indicated by the monotonically increasing RMS values for both variables. As noted earlier, this is consistent with experimental observations, where frost deposition varies along both the fins and the tubes.

Both temperature and frost thickness increase monotonically on average with an RMS value that also increases monotonically with time. At the start of the simulation, the entire surface of the fins and tubes is held at a constant temperature, without any frost deposits. As the frost begins to deposit on the surfaces, and in particular on the fins, variations in thickness and surface temperature are observed, as shown in panels c and d in figure 5. Our focus in this study is on early time evolution, whereby the frost deposition amounts to a few per cent of the fin-to-fin separation of the heat exchanger. By the end of the simulation at τt=1500, the maximum temperature of the frost surface has increased by about 16% of the difference between the bulk temperature and the fin/tube temperature, while the average temperature has increased to about 8%. Taking into account the applied bulk temperature of $T_{b}^{*}=295$ K and fin-and-tube temperature of $T_{p}^{*}=263$ K, an increase in the temperature difference of 8% corresponds to a frost surface temperature of around 266 K. Similarly, a 16% increase in the temperature difference corresponds to a frost surface temperature of around 268 K. By the end of the simulation in case 3, the average thickness of the frost on the fins is approximately 4% of the fin-to-fin separation distance. The maximum and minimum ensemble average values are around 6 and 2%, respectively. During the time period considered, the frost accumulation rate is nearly linear, with the RMS value also increasing linearly. In contrast, the rate of increase in frost surface temperature slows over time.

**Figure 6.**
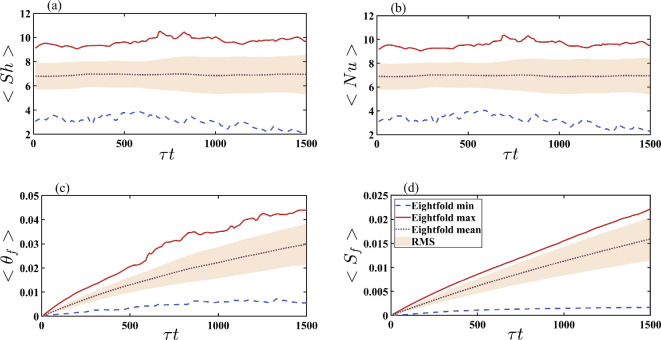
Minimum, maximum, mean and RMS values from the eightfold average on the tubes for (a) Sherwood number, (b) Nusselt number, (c) frost surface temperature and (d) frost thickness. τ=1000 is the slow-time acceleration factor, and t represents the non-dimensional time.

Figure 6 shows the frost layer statistics for the tubes again in terms of the Sherwood number (panel a), Nusselt number (panel b), frost surface temperature (panel c) and frost thickness (panel d). The average frost thickness on the tubes corresponds to about 1.5% of the half fin-to-fin separation distance by the end of the simulation, as indicated in panel d. This is roughly 2.5 times smaller than what was observed on the fins in figure 5d. Since the frost build-up is much smaller, the frost surface temperature is minimally affected, remaining marginally above the tube surface temperature as seen in panel c. The average frost surface temperature is about one-third of what is observed on the fins, which is also true for the maximum and minimum frost surface temperatures. The RMS value is, however, much larger than on the fins, being equivalent to about 30% of the mean. This large variation is due to the difference between the windward and leeward sides of the tubes, which is again consistent with experimental observations [22]. The average Nusselt and Sherwood numbers also remain constant for the duration of the simulation attaining a value that is about a third of what is observed on the fins, with an RMS value of around 40%. The maximum and minimum values fluctuate around 9 and 3, respectively.

**Figure 7.**
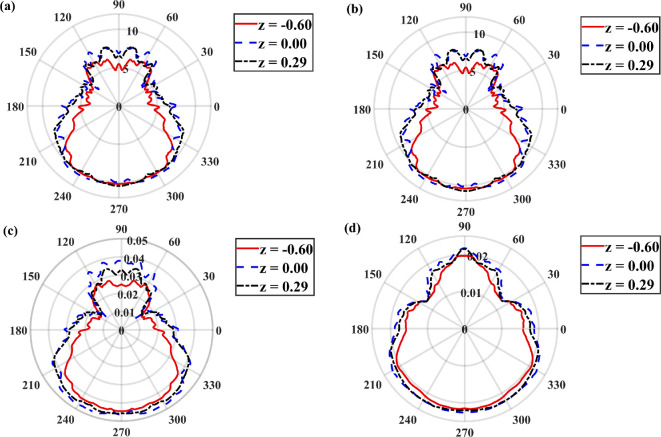
Polar plots of (a) Nusselt number, (b) Sherwood number, (c) frost surface temperature and (d) frost thickness on the tubes at three different z positions. The data correspond to case 3 at τt=1500 and is eightfold averaged.

As seen from the contour plots in figures 4 and 6, the frost exhibits significant variations on the tubes. This variation may be examined as seen in figure 7, where we plot the eightfold-averaged azimuthal variation of the Nusselt number (panel a), Sherwood number (panel b), frost surface temperature (panel c) and frost thickness (panel d) on the tubes. The plots correspond to three different vertical positions, namely z=−0.6, 0 and 0.29. The flow is again oriented such that the windward and leeward sides align with ϕ=3π/2 and ϕ=π/2, respectively. These results correspond to case 3 at τt=1500. The behaviour of the frost layer across the three different vertical positions is similar, indicating little variation along the vertical direction of the tubes. However, there are noticeable differences between the windward and the leeward sides. The highest values for all variables occur on the windward side for ϕ values ranging from 210 to 330 degrees, i.e. 7π/6≤ϕ≤11π/6. However, interestingly, the lowest values do not occur on the leeward side but rather near the edge of the tubes where the flow begins to separate. This behaviour is observed at different z locations. The temperature and water vapour fraction are passive scalars, and as such, the recirculation regions that form due to flow separation result in reduced heat and mass transfer rates, which are reflected in the smaller Nusselt and Sherwood numbers. As the Nusselt and Sherwood numbers remain consistently small, so do the frost thickness and frost surface temperature, resulting in a pear shape for frost deposition around the tubes. The minimum thickness of the frost layer occurs around ϕ=π/6 and ϕ=5π/6 measuring approximately half the frost thickness on the windward side at ϕ=3π/2.

### (d) Effect of Reynolds number

**Figure 8.**
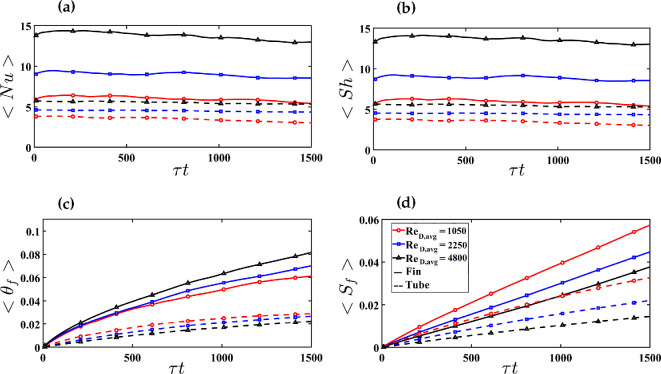
Temporal evolution of the spatially averaged values over the fins (dashed lines) and tubes (solid lines) for (a) Nusselt number, (b) Sherwood number, (c) frost surface temperature and (d) frost thickness over time.

Finned-tube heat exchangers can operate at various flow rates. For a given geometry, an increase in the bulk velocity leads to a corresponding increase in the bulk Reynolds number. As the Reynolds number increases, the turbulence intensity also grows, enhancing both mass transfer (represented by the Sherwood number) and heat transfer (represented by the Nusselt number). Figure 8 shows the average Nusselt and Sherwood numbers, the frost surface temperature and the frost thickness on the fins (solid lines) and tubes (dashed lines) from the three simulations, which correspond to $Re_{D,\text{avg}}=1050$ for case 1 (red), $Re_{D,\text{avg}}=2250$ for case 2 (blue) and $Re_{D,\text{avg}}=4800$ for case 3 (black). The trend in the Sherwood and Nusselt numbers is as expected, with larger values for the fins compared to those of the tubes, as well as larger values for larger Reynolds numbers. For example, in the case of the tubes, the Nusselt and Sherwood numbers increase by about a factor of 2 as the Reynolds number increases by about a factor of 4 from 1050 to 4800. For the fins, the average Nusselt and Sherwood numbers increase from approximately 6 to 14 as $Re_{D,\text{avg}}$ increases from 1050 to 4800.

The observed trends in frost surface temperature and frost thickness might initially seem counterintuitive. Specifically, the thickness of the frost layer decreases with increasing Reynolds number ($Re_{D,\text{avg}}$), while the surface temperature exhibits different trends on the fins and tubes, whereby on the tubes, the average surface temperature decreases with Reynolds number, while on the fins, the average surface temperature increases with Reynolds number. This behaviour arises from the non-dimensionalization process. Comparing the frost surface temperature or frost thickness at the same non-dimensional time for different Reynolds numbers does not imply a comparison at the same dimensional time. In the present simulations, increasing the Reynolds number corresponds to a higher bulk velocity, while the geometry and characteristic length scale remain constant. Consequently, the time scale for each simulation differs because it is inversely proportional to the velocity scale and the Reynolds number. For example, the time scale for the highest Reynolds number in case 3 ($Re_{D,\text{avg}}=4800$) is approximately half that of case 2 and one-quarter that of case 1. Thus, $\left\langle S_{f}\right\rangle$ at τt=800 in case 3 corresponds to the same dimensional time as τt≈400 in case 2 and τt≈200 in case 1. When evaluated at the same dimensional time, the average temperature and thickness at the frost surface increase with Reynolds number. This Reynolds number scaling also influences the plots of frost surface temperature. Although the frost surface temperature on the fin grows faster with non-dimensional time at higher $Re_{D,\text{avg}}$*,* the trend reverses on the tube. However, at comparable dimensional times, the frost thickness and frost surface temperature increase with Reynolds number along both the fin-and-tube surfaces. In contrast, this issue does not arise for the average Nusselt and Sherwood numbers, which remain largely unchanged throughout the simulations.

**Figure 9.**
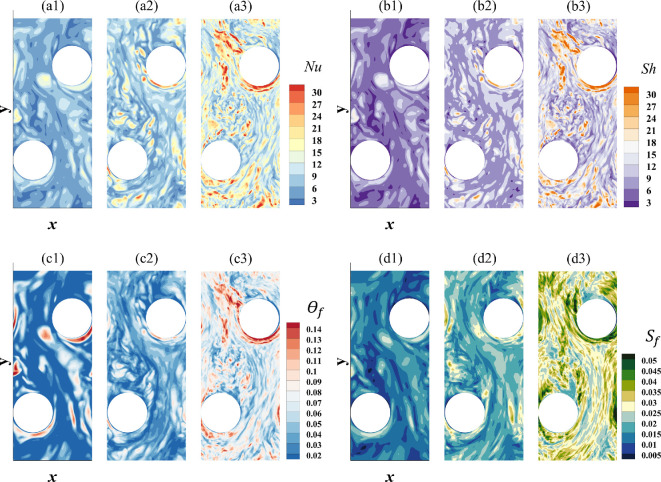
(a) Nusselt number, (b) Sherwood number, (c) frost surface temperature and (d) frost thickness from cases 1, 2 and 3 on the bottom plate (fin) at τt=300, 600 and 1200, respectively.

Figure 9 presents the contours of the Nusselt and Sherwood numbers, the frost surface temperature and the frost thickness on the bottom fin at τt=300, 600 and 1200 for cases 1, 2 and 3, respectively. The selected time instances ensure a meaningful comparison by aligning the dimensional time across the three Reynolds numbers. The flow remains turbulent in all three cases, as evident from the contour plots. However, finer turbulent scales emerge with an increase in the Reynolds number. Consequently, the macroscale frost surface appears smoother in case 1 compared to case 2 and in case 2 compared to case 3. A similar trend is observed in the variation of the frost surface temperature with smaller Reynolds numbers, resulting in reduced variation and a more uniform surface temperature. Furthermore, the plots clearly show that frost accumulates more rapidly at higher Reynolds numbers, with the frost surface temperature being higher, indicating a denser frost layer.

**Figure 10.**
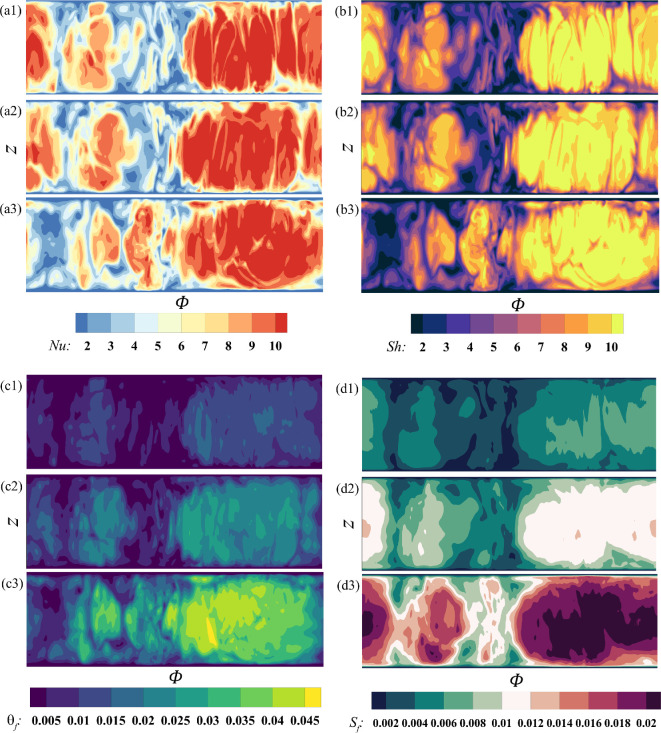
(a) Nusselt number, (b) Sherwood number, (c) frost surface temperature and (d) frost thickness from cases 1, 2 and 3 on the upstream tube at τt=300, 600 and 1200, respectively.

**Figure 11.**
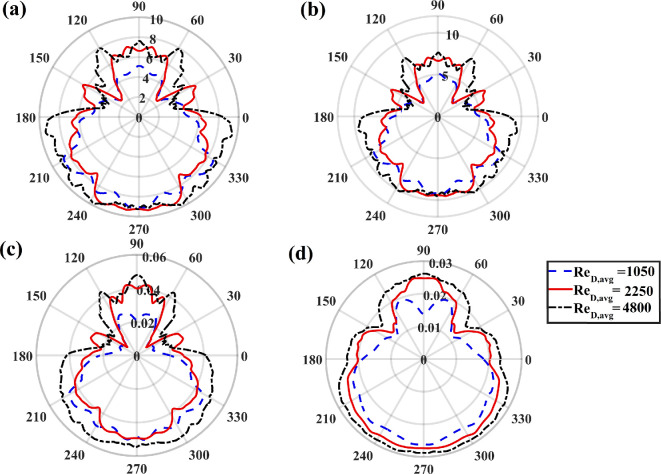
Polar plots of (a) Nusselt number, (b) Sherwood number, (c) frost surface temperature and (d) frost thickness on the tubes at three different Reynolds numbers. The data correspond to case 3 at τt=1500 and is eightfold averaged.

The behaviour observed on the fins in figure 9 is similarly evident on the tubes in figure 10, where contours of the Nusselt and Sherwood numbers, the frost surface temperature and the frost thickness are presented for the upstream tube at τt=300, 600 and 1200 for cases 1, 2 and 3, respectively. As with the fins, the contours exhibit smoother profiles at lower Reynolds numbers; however, the differences between cases are less pronounced. For example, the frost thickness and surface temperature vary within comparable ranges in the three cases. However, the azimuthal variation remains consistent in all cases as the Reynolds number varies. In figure 11, we present the eightfold-averaged azimuthal variation of the Nusselt number (panel a), Sherwood number (panel b), frost surface temperature (panel c) and frost thickness (panel d) on the tubes at τt=1500. The plots correspond to the following three different Reynolds numbers: $Re_{D,\text{avg}}=1050$, 2250 and 4800. We observe similar trends in the different cases. For example, the variables attain their largest values on the windward side and their smallest values between the equatorial points (i.e. $\phi ={0,180}^{\circ }$) and the downward stagnation point (i.e. $\phi =90^{\circ }$). Nonetheless, we observe for the largest Reynolds number that the values of the Nusselt and Sherwood numbers remain elevated on the entire windward side. This is in contrast with the two lower Reynolds number cases, whereby the maximum value of the Nusselt and Sherwood numbers is observed near the upstream stagnation point, but that value noticeably decreases near the equatorial points.

## 4. Conclusions

We conducted turbulence-resolving simulations to examine frost accumulation in fin-and-tube heat exchangers typical of those used as freezer coils in industrial refrigeration applications. The simulations are dynamically two-way coupled employing the immersed boundary method, by which the frost accumulation affects the airflow velocity, temperature and water vapour fraction, and, at the same time, the airflow through the surface gradients of temperature and water vapour fraction affects the evolution of the frost layer on both the fins and the tubes. We implemented a slow-time acceleration technique to accelerate frost accumulation and reduce the computational cost of the simulations. The slow-time acceleration is needed due to the vastly different time scales between the fast turbulent airflow and the slow frost accumulation process.

By examining the Nusselt and Sherwood numbers, we observe temporal and spatial variation, whereby the averaged scaled Nusselt and Sherwood numbers remain relatively unchanged for the duration of the simulation, being largest on the fins compared to the tubes and on the windward side of the tubes compared to the leeward side. In particular, the Nusselt and Sherwood numbers are about a factor of 2 to 3 larger on the fins, which results in frost accumulating at an equivalent faster rate on the fins. This behaviour has also been reported in laboratory experiments in which frost was observed to accumulate faster on the fins (compared to the tubes) and on the windward side of the tubes compared to the leeward side. Due to the turbulent nature of the flow, the local Nusselt and Sherwood numbers show significant spatial and temporal variations, with their maxima—approximately twice the average—exceeding the minima by more than an order of magnitude. This behaviour is true for both the fins and the tubes. Furthermore, the RMS variation is observed to be around 30 and 50% of the mean for the fins and tubes, respectively. The spatial variation of the Nusselt and Sherwood numbers is reflected in the frost thickness and surface temperature, whereby both variables exhibit strong variations.

Fin-and-tube heat exchangers can operate across a range of flow rates, with higher flow rates corresponding to increased bulk Reynolds numbers. We performed three simulations using identical geometry, spatial and temporal resolutions, differing only in Reynolds numbers, all within the turbulent flow regime. The results show that as the Reynolds number increases, the heat and mass transfer rates are enhanced. However, spatial gradients diminish at lower Reynolds numbers, resulting in smoother profiles for the frost thickness and frost surface temperature. Despite improved heat transfer at higher Reynolds numbers, frost accumulation increases, which can counteract these benefits over time by forming an insulating layer. In our simulations, the Nusselt and Sherwood numbers, representing heat and mass transfer rates, doubled as the bulk Reynolds number increased fourfold, from 1050 to 4800—values representative of realistic operating conditions for fin-and-tube heat exchangers.

## Data Availability

All data are included in the paper.

## Appendix A. Enhancement factor

The polynomial form of the enhancement factor is given by

(A 1)
$$\begin{array}{r} \epsilon^{*}=d_{0}^{*}+d_{1}^{*}(T_{f}^{*}-273.15)+d_{2}^{*}(T_{f}^{*}-273.15{)}^{2}+d_{3}^{*}(T_{f}^{*}-273.15{)}^{3}+\\ d_{4}^{*}(T_{f}^{*}-273.15{)}^{4}+d_{5}^{*}(T_{f}^{*}-273.15{)}^{5}\;. \end{array}$$

The partial pressure of water vapour over ice, $p_{f}^{*}$ in Pa, is calculated as the saturation pressure of water vapour over ice at the frost surface temperature $T_{f}^{*}$. Following the formulation provided in [43], it is expressed as:

(A 2)
$$p_{f}^{*}=\exp\left(a_{0}^{*}/T_{f}^{*}+a_{1}^{*}+a_{2}^{*}T_{f}^{*}+a_{3}^{*}T_{f}^{{*}^{2}}+a_{4}^{*}T_{f}^{{*}^{3}}+a_{5}^{*}T_{f}^{{*}^{4}}+a_{6}^{*}\ln T_{f}^{*}\right).$$

where $a_{0}^{*},a_{1}^{*},\ldots ,a_{6}^{*}$ are empirical coefficients derived for the temperature range $173.15\;\text{K}\leq T_{f}^{*}\leq 273.15\;\text{K}$. The numerical values of the coefficients in equations (A 1) and (A 2) are as follows: $d_{0}^{*}=1.00391723$, $d_{1}^{*}=-7.8220553\times 10^{-6}$, $d_{2}^{*}=6.94681594\times 10^{-7}$, $d_{3}^{*}=3.040587\times 10^{-9}$, $d_{4}^{*}=-2.7852\times 10^{-11}$, $d_{5}^{*}=-5.656\times 10^{-13}$, $a_{0}^{*}=-0.56745359\times 10^{4}$, $a_{1}^{*}=6.3925247$, $a_{2}^{*}=-0.9677843\times 10^{-2}$, $a_{3}^{*}=-0.62215701\times 10^{-6}$, $a_{4}^{*}=0.20747825\times 10^{-8}$, $a_{5}^{*}=-0.9484024\times 10^{-12}$ and $a_{6}^{*}=0.41635019\times 10^{1}$.

While many empirical relations exist for these properties [44–47], the selected set provides the best match to experimental data within the temperature and velocity ranges considered in this study.
